# Gallager Exponent Analysis of Coherent MIMO FSO Systems over Gamma-Gamma Turbulence Channels

**DOI:** 10.3390/e22111245

**Published:** 2020-11-01

**Authors:** Maoke Miao, Xiaofeng Li

**Affiliations:** School of Aeronautics and Astronautics, University of Electronic Science and Technology of China, Chengdu 610054, China; mmk1993@std.uestc.edu.cn

**Keywords:** gamma–gamma turbulence channels, Hadamard inequality, random coding exponent, ergodic capacity, expurgated exponent, STBC

## Abstract

This paper studies the Gallager’s exponent for coherent multiple-input multiple-output (MIMO) free space optical (FSO) communication systems over gamma–gamma turbulence channels. We assume that the perfect channel state information (CSI) is known at the receiver, while the transmitter has no CSI and equal power is allocated to all of the transmit apertures. Through the use of Hadamard inequality, the upper bound of the random coding exponent, the ergodic capacity and the expurgated exponent are derived over gamma–gamma fading channels. In the high signal-to-noise ratio (SNR) regime, simpler closed-form upper bound expressions are presented to obtain further insights into the effects of the system parameters. In particular, we found that the effects of small and large-scale fading are decoupled for the ergodic capacity upper bound in the high SNR regime. Finally, a detailed analysis of Gallager’s exponents for space-time block code (STBC) MIMO systems is discussed. Monte Carlo simulation results are provided to verify the tightness of the proposed bounds.

## 1. Introduction

Over the past few years, the ergodic capacity has been intensively investigated over various types of fading channels for single-input single-output (SISO) and multiple-input multiple-output (MIMO) systems, since it determines the fundamental limit on achievable information rates of communication systems [1,2,3,4,5]. However, considering that this metric can not be sufficient to reflect the limits of communication systems, a stronger form of the channel coding theorem has been pursued to describe the relation among the error probability $P_{e}$, codeword length *N* and information rate *R*. Specifically, it is shown that for any rate less than the channel capacity, the error probability for the optimal block code satisfies [6,7]

(1) $$E\left(R\right)≜\underset{N\to \infty }{lim sup}\frac{-\ln P_{e}^{opt}\left(R,N\right)}{N}$$

where E(R) is defined as a reliability function or error exponent and is typically difficult to obtain. According to Equation (1), it can be observed that the error probability approaches zero as the codeword length tends to infinity for a rate below the channel capacity. However, it is difficult to find the supremum of the function E(R) through this expression. The classical lower bound of the error exponent, known as the random coding error exponent or Gallager’s exponent [8], is easily computable and has been used to estimate the codeword length required to achieve a prescribed error probability.

Since then, a large amount of research has investigated the random coding error exponent for single-input single-output (SISO) and single-input multiple-output (SIMO) flat-fading channels with average or peak power constraints [9,10]. In [11], assuming that the channel state information (CSI) is perfectly known at the receiver, a random coding error exponent for the Rayleigh fading channel with different types of diversity methods was discussed. Then, these results were later extended to the multiple-input multiple-output (MIMO) Rayleigh block fading channel in [12], which demonstrates that having more transmitted antennas than the piecewise-constant in blocks of *T* time samples does not increase the random coding error exponent and the fading coherence time plays a fundamental role in the error exponent. Moreover, a detailed analysis for the effects of channel coherence time and spatial fading correlation on the MIMO error exponent for Rayleigh channel was presented in [7]. The relationship between the probability error, information rate, codeword length and signal-to-noise ratio (SNR) for fast Rayleigh fading MIMO-ARQ channels was examined in [13]. Additionally, Gallager’s exponent for the generalized fading MIMO channels was studied in [14,15]; the space-time clock coded (STBC) technique was employed. More recently, closed-form expressions of Gallager random coding and expurgated error exponents for multi-keyhole MIMO channels were discussed in [16]. It should be noted, however, that the closed-form expressions of random coding exponent can be carried out only for the MIMO complex Gaussian channels because the joint eigenvalue distribution of them can be mathematically formulated according to the well-known Wishart matrix theory. Additionally, all the results presented above are limited in the radio frequency (RF) communication. As far as we know, no prior work has been carried out to study this important metric in a novel wireless communication technique, i.e., free space optical (FSO) communication, where the gamma–gamma fading channel is popularly considered due to its excellent agreement with experimental data for a range of turbulence conditions [17].

In this paper, we study the Gallager’s exponent of MIMO gamma–gamma block fading channels. The Gaussian inputs subject to the average power constraint and perfect CSI at the receiver are considered. In particular, the analytical upper bound of Gallager’s exponent is established by virtue of Hadamard inequality, which avoids the need for the eigenvalue distribution of the channel matrix $HH^{†}$. The proposed upper bound is developed in terms of MeijerG function and it can be easily evaluated and efficiently programmed in most standard software packages (e.g., MATLAB or MATHEMATICA). The asymptotic bound performance in the high-SNR regime is obtained in order to assess the impacts of system parameters readily. We also elaborate on the ergodic capacity, which can be directly derived from Gallager’s exponent. Moreover, we find that the effects of small and large-scale fading are decoupled at high SNRs. The expurgated exponent upper bound for MIMO systems over the gamma–gamma block fading channel is also studied in this paper. For the sake of completeness, these previous results are then extended to STBC MIMO systems.

The remainder of the paper is organized as follows. In Section 2, we describe the coherent MIMO FSO system model and introduce Gallager’s exponent. In Section 3, using Hadamard inequality, the analytical upper bounds for Gallager’s exponent, ergodic capacity and the expurgated exponent are derived and analyzed in detail. Gallager’s results of STBC over MIMO gamma–gamma block fading channels are presented in Section 4. Finally, we conclude the paper in Section 5.

## 2. The System and Gallager’s Exponent

### 2.1. System Model

**Figure 1:**
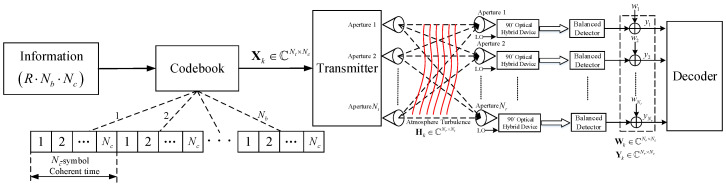
Coherent multiple-input multiple-output (MIMO) block-fading channel with $N_{t}$ transmission and $N_{r}$ receiver apertures over $N_{b}$ independent $N_{c}$-symbol coherence intervals.

A system block diagram of block-fading MIMO FSO systems using the coherent detection is shown in Figure 1. For an observation interval of $N_{b}N_{c}$ symbol periods, the channel fading is memoryless for each $N_{c}$ symbol. All the $N_{b}N_{c}$ symbols are simultaneously transmitted through the $N_{t}$ transmit apertures.

The received electric field at the *n*-th, $n\in \{1,2,\cdots ,N_{r}\}$, receiver aperture from the *m*-th, $m\in \{1,2,\cdots ,N_{t}\}$ transmit aperture, is given by

(2) $$E_{mn}\left(t\right)=\sqrt{P_{mn}}A_{s,m}\exp\left(j\left(\omega t+\phi_{mn}+\phi_{s,m}\right)\right)$$

where $P_{mn}$ denotes the received power and is subject to the optical scintillation; ω is the optical carrier frequency of the transmit signal laser; $\phi_{mn}$ represents the overall phase noise from the *m*-th transmit aperture to *n*-th receiver aperture and can be modeled as a Wiener process [18]; and $A_{s,m}$ and $\phi_{s,m}$ are the encoded amplitude information and encoded phase information respectively. The electric field of the local oscillator (LO) can be expressed as

(3) $$E_{LO}\left(t\right)=\sqrt{P_{LO}}\exp\left(j\left(\omega_{LO}t+\phi_{LO}\right)\right)$$

where $P_{LO}$ is the power of the LO, $\omega_{LO}$ denotes the optical carrier frequency of the LO, and $\phi_{LO}$ represents the phase noise of the LO.

Using the $2\times 4\;90^{\circ }$ optical hybrid mixer and two pairs of balanced photodetectors [19], we can derive the four output photocurrents as

(4) $$\left\{\begin{array}{cc} i_{1}\left(t\right) & =\frac{1}{4}\cdot R_{oe}{\left|E_{LO}\left(t\right)+\sum_{m=1}^{N_{t}}E_{mn}\left(t\right)\right|}^{2}\\ i_{2}\left(t\right) & =\frac{1}{4}\cdot R_{oe}{\left|jE_{LO}\left(t\right)+\sum_{m=1}^{N_{t}}E_{mn}\left(t\right)\right|}^{2}\\ i_{3}\left(t\right) & =\frac{1}{4}\cdot R_{oe}{\left|-E_{LO}\left(t\right)+\sum_{m=1}^{N_{t}}E_{mn}\left(t\right)\right|}^{2}\\ i_{4}\left(t\right) & =\frac{1}{4}\cdot R_{oe}{\left|-jE_{LO}\left(t\right)+\sum_{m=1}^{N_{t}}E_{mn}\left(t\right)\right|}^{2} \end{array}\right.$$

where $R_{oe}$ denotes the photodiode responsibility and $i_{1}\left(t\right),i_{2}\left(t\right),i_{3}\left(t\right),i_{4}\left(t\right)$ denote the $0^{\circ }$, $90^{\circ }$, $180^{\circ }$ and $270^{\circ }$ respectively. Note that we have assumed that carrier synchronization is perfect in the receiver. The in-phase and quadrature signals can be obtained as

(5) $$\begin{array}{cc} i_{I}\left(t\right) & =i_{1}\left(t\right)-i_{3}\left(t\right)=\sum_{m=1}^{N_{t}}\eta_{mn}A_{s,m}\cos\left(\phi_{mn}-\phi_{LO}+\phi_{s,m}\right)\\ i_{Q}\left(t\right) & =i_{2}\left(t\right)-i_{4}\left(t\right)=\sum_{m=1}^{N_{t}}\eta_{mn}A_{s,m}\sin\left(\phi_{mn}-\phi_{LO}+\phi_{s,m}\right). \end{array}$$

Thus, the *n*-th received signal at the input of the decoder can be expressed as

(6) $$y_{n}\left(t\right)=i_{I}\left(t\right)+ji_{Q}\left(t\right)+w_{n}\left(t\right)=\sum_{m=1}^{N_{t}}\eta_{mn}A_{s,m}\exp\left(j\left(\Delta \phi_{mn}+\phi_{s,m}\right)\right)+w_{n}\left(t\right)$$

where $\Delta \phi =\phi_{mn}-\phi_{LO}$ is assumed to be uniformly distributed between 0 and 2π for convenience. The signal $w_{n}\left(t\right)$ is zero-mean complex Gaussian noise with independent, equal variance real and imaginary parts. According to Equation (6), the term $h_{mn}=\eta_{mn}\exp\left(j\Delta \phi_{mn}\right)$ can be regarded as the channel fading and $\left|\right|h_{mn}{\left|\right|}^{2}=I_{mn}$ follows the gamma–gamma distribution given by Equation (8) when the intensity of $h_{mn}$ is normalized.

Therefore, based on the above information, the received signal at the input of the decoder for the *k*-th coherence interval can be expressed as

(7) $$Y_{k}=H_{k}X_{k}+W_{k}$$

where $X_{k}\in C^{N_{t}\times N_{c}}$ represents transmitted signal matrices, $H_{k}\in C^{N_{r}\times N_{t}}$ is the channel gain matrices and $W_{k}∼N_{N_{t},N_{c}}\left(0,N_{0}I_{N_{r}},I_{N_{c}}\right)\in C^{N_{r}\times N_{c}}$ is additive white Gaussian noise (AWGN). The entries of $H_{k}$ are denoted by $h_{k,i}\left(i=1,2,\cdots ,N_{r}N_{t}\right)$ and are assumed to be statistically independent, of which the amplitude and phase follow Generalized-*K* ($K_{G}$) and uniform distribution respectively [20,21]. According to [22], the so-called gamma–gamma distribution considered here is equivalent to the squared $K_{G}$ distribution, which is given by

(8) $$f\left(I\right)=\frac{2{\left(ab\right)}^{\frac{a+b}{2}}}{\Gamma \left(a\right)\Gamma \left(b\right)\Omega^{\frac{a+b}{2}}}I^{\frac{a+b}{2}-1}K_{a-b}\left[2\sqrt{\frac{ab}{\Omega }I}\right]$$

where $K_{v}\left(\cdot \right)$ denotes the modified Bessel function of order *v*; $\Gamma \left[\cdot \right]$ is the Gamma function; and Ω is related with mean, i.e., $E\left[I\right]=\Omega$ with E denoting expectation. The large-scale fading a>0 and small-scale fading b>0 are the distribution shaping parameters that can be expressed as

(9) $$a={\left[\exp\left(\frac{0.49\sigma_{R}^{2}}{\left(1+0.18d^{2}+0.56\sigma_{R}^{6/5}\right)}\right)-1\right]}^{-1}$$

and

(10) $$b={\left[\exp\left(\frac{0.51\sigma_{R}^{2}}{{\left(1+0.9d^{2}+0.62\sigma_{R}^{6/5}\right)}^{7/6}}\right)-1\right]}^{-1}$$

where $\sigma_{R}^{2}=1.23C_{n}^{2}k^{7/6}L^{11/6}$ is the Rytov variance and $d=\sqrt{kD^{2}/4L}$ with k=2π/λ is the optical wave number, *L* is the length of the optical link, *D* denotes the receiver’s aperture diameter and $C_{n}^{2}$ is the refractive index structure constant that can be used as a measure of the strength of the turbulence and varies from $10^{-17}\;m^{-2/3}$ for weak turbulence to $10^{-13}\;m^{-2/3}$ for strong turbulence. The typical parameters for wavelength, receiver’s aperture and the length of the optical link were set to be 850 nm, 0.01 m and 1000 m respectively [23].

Moreover, the input signal matrix $X_{k}$ is assumed to satisfy an average power constraint, i.e.,

(11) $$\frac{1}{N_{c}}E\left[\mathrm{tr}\left(X_{k}X_{k}^{†}\right)\right]=\mathrm{tr}\left(Q\right)\leq P$$

where Q represents the $N_{t}\times N_{t}$ positive semidefinite matrix and P is the total transmit power over $N_{t}$ transmit apertures. In the later analysis, we define the random matrix $\Theta \in C^{m\times m}$ as

(12) $$\Theta ≜\left\{\begin{array}{cc} H_{k}H_{k}^{†}, & \;\mathrm{if}\;N_{r}\leq N_{t}\\ H_{k}^{†}H_{k}, & \;\mathrm{otherwise}\; \end{array}\right.$$

and $s≜\min\left\{N_{t},N_{r}\right\}$, $t≜\max\left\{N_{t},N_{r}\right\}$.

Specifically, note that the MIMO channel can be collapsed into a single channel for each symbol when employing the space-time block codes (STBC) technique [24]. Thus, the effective output symbol SNR is given by

(13) $$\gamma_{0}=\frac{P}{R_{c}N_{0}}\left|\right|H_{k}{\left|\right|}_{F}^{2}$$

where $R_{c},||\cdot {\left|\right|}_{F}$ are the code rate and Frobenius norm respectively. Without loss of generality, full-rate STBC is assumed such that $R_{c}=1$. We can omit the index *k* for channels memoryless and stationary over each coherence time interval.

### 2.2. Gallager’s Exponent

In this subsection, we present a detailed description of Gallager’s exponent, which gives the upper bound of error probability with maximum-likelihood (ML) decoding for a channel with continuous inputs and outputs. Additionally, Gallager’s exponent provides us a significant look into the reliability-rate tradeoff in MIMO communication. Specifically, it is shown that the diversity-multiplexing tradeoff of MIMO channels can be regarded as a special case of the reliability-rate tradeoff in the high SNR regime [25].

(1) *Random coding exponent*: The random coding bound on the error probability of ML decoding was developed in [8], which is given by

(14) $$P_{e}\leq {\left(\frac{2e^{r\delta }}{\xi }\right)}^{2}e^{-N_{b}N_{c}E_{r}\left(p_{X}\left(X\right),R,N_{c}\right)}$$

where

(15) $$\begin{array}{cc} \xi & \approx \frac{\delta }{\sqrt{2\pi N_{b}\sigma_{\xi }^{2}}}\\ \sigma_{\xi }^{2} & =\int_{X}\left[\mathrm{tr}\left(XX^{†}\right)-N_{c}P\right]p_{X}\left(X\right)dX. \end{array}$$

The above bound involves a number of random parameters, namely r,δ≥0 and input distribution $p_{X}\left(X\right)$.

The random coding exponent $E_{r}\left(p_{X}\left(X\right),R,N_{c}\right)$ in Equation (14) is defined as

(16) $$\begin{array}{c} E_{r}\left(p_{X}\left(X\right),R,N_{c}\right)=\max_{0\leq \rho \leq 1,p_{X}\left(X\right)}\left\{\max_{r\geq 0}E_{0}\left(p_{X}\left(X\right),\rho ,r,N_{c}\right)-\rho R\right\} \end{array}$$

with $E_{0}\left(p_{X}\left(X\right),\rho ,r,N_{c}\right)$ shown in Equation (17).

(17) $$E_{0}\left(p_{X}\left(X\right),\rho ,r,N_{c}\right)=-\frac{1}{N_{c}}\ln\left\{\int_{H}p_{H}\left(H\right)\int_{Y}{\left(\int_{X}p_{X}\left(X\right)e^{r\left[\mathrm{tr}\left(XX^{†}\right)-N_{c}P\right]}p{\left(Y|X,H\right)}^{1/(1+\rho )}dX\right)}^{1+\rho }dYdH\right\}.$$

Generally, optimizing the input distribution $p_{X}\left(X\right)$ for the maximization error exponent is a difficult task. However, the assumption of capacity-achieving Gaussian distribution for $p_{X}\left(X\right)$ that is subject to the power constraint can make the problem analytically tractable, though it is valid only if the rate *R* approaches the channel capacity. As such, $p_{X}\left(X\right)$ is given by

(18) $$p_{X}\left(X\right)=\pi^{-N_{t}N_{c}}\det{\left(Q\right)}^{-N_{c}}\mathrm{etr}\left(-Q^{-1}XX^{†}\right)$$

where $\mathrm{etr}\left(\cdot \right)=e^{tr\left(\cdot \right)}$. Substituting Equation (18) into Equation (17), we then have ([7], (Proposition 1))

(19) $$\begin{array}{c} E_{0,G}\left(Q,\rho ,r,N_{c}\right)=rP\left(1+\rho \right)+\left(1+\rho \right)\ln\det\left(I_{n_{T}}-rQ\right)-\frac{1}{N_{c}}\ln E\left\{\det{\left(I_{n_{R}}+\frac{H{\left(Q^{-1}-rI_{n_{T}}\right)}^{-1}H^{†}}{N_{0}\left(1+\rho \right)}\right)}^{-N_{c}\rho }\right\} \end{array}$$

**Proposition** **1.** *Equation (16) can be maximized with equal transmit power when the Gaussian inputs are assumed, i.e., $Q=\frac{P}{N_{t}}I_{N_{t}}$.*

**Proof.**  A proof is given in Appendix A. □

For the case of equal power allocation to each transmit aperture, Equation (19) can be further reduced to

(20) $$\begin{array}{c} {\tilde{E}}_{0}\left(\rho ,\beta ,N_{c}\right)=E_{0,G}\left(\frac{P}{N_{t}}I_{N_{t}},\rho ,r,N_{c}\right)\\ =\underset{A(\rho ,\beta )}{\underset{︸}{\left(1+\rho \right)\left(N_{t}-\beta \right)+N_{t}\left(1+\rho \right)\ln\left(\beta /N_{t}\right)}}-\frac{1}{N_{c}}\ln\left(E\left\{\det{\left(I_{s}+\frac{\gamma \Theta }{\beta (1+\rho )}\right)}^{-N_{c}\rho }\right\}\right) \end{array}$$

after some algebraic manipulations. Then the random coding exponent in Equation (16) becomes

(21) $$E_{r}\left(R,N_{c}\right)=\max_{0\leq \rho \leq 1}\left\{\max_{0\leq \beta \leq N_{t}}{\tilde{E}}_{0}\left(\rho ,\beta ,N_{c}\right)-\rho R\right\}.$$

As shown in [7], a new upper bound on the error probability is given by

(22) $$P_{e}\leq \frac{8\pi }{N_{t}}{\left(N_{t}-\beta^{*}\left(\rho \right)\right)}^{2}N_{b}N_{c}e^{\left(2-N_{b}N_{c}E_{r}\left(R,N_{c}\right)\right)}$$

which will be used for estimating the required codeword length $L=N_{c}\left\lceil N_{b}\right\rceil$, given rate *R* and prescribed $P_{e}$ in the latter, where $\left\lceil \cdot \right\rceil$ denotes the smallest integer larger than or equal to an enclosed quantity. $\beta^{*}\left(\rho \right)$ in Equation (22) denotes the value β that maximizes ${\tilde{E}}_{0}\left(\rho ,\beta ,N_{c}\right)$ defined in Equation (20) for each ρ, and is in the range $0<\beta \leq N_{t}$.

(2) *Ergodic capacity*: According to [7], the information rate *R* can be expressed as

(23) $$R=\frac{\partial {\tilde{E}}_{0}\left(\rho ,\beta^{*}\left(\rho \right),N_{c}\right)}{\partial \rho }.$$

Note that *R* becomes identical to the Shannon (ergodic) capacity 〈C〉 defined in [1] when ρ=0 and $\beta =N_{t}$, such that

(24) $$\begin{array}{cc} \langle C\rangle & =E\left\{\ln\det\left(I_{s}+\frac{\gamma }{N_{t}}\Theta \right)\right\}={\left.\frac{\partial {\tilde{E}}_{0}\left(\rho ,\beta^{*}\left(\rho \right),N_{c}\right)}{\partial \rho }\right|}_{\rho =0,\beta^{*}\left(0\right)=N_{t}} \end{array}$$

where $\gamma =\frac{P}{N_{0}}$ denotes the SNR. The above formula indicates the relation between Gallager exponent and Shannon capacity.

(3) *Expurgated exponent*: It has been shown in [8] that the random coding exponent is defined by choosing the codeword independently according to input distribution $p_{X}\left(X\right)$ In other words, the good and bad codewords contribute equally to the overall average error probability. However, the poor codewords dominate the average error probability and have an adverse effect on the random coding exponent. Thus, the random coding exponent can be improved by expurgating poor codewords form the ensemble and is given by

(25) $$E_{ex}\left(p_{X}\left(X\right),R,N_{c}\right)=\max_{\rho \geq 1}\left(\max_{r\geq 0}E_{x}\left(p_{X}\left(X\right),\rho ,r,N_{c}\right)-\rho R\right)$$

with $E_{x}\left(p_{X}\left(X\right),\rho ,r,N_{c}\right)$ defined in Equation (26) as follows

(26) $$\begin{array}{cc} E_{x}\left(p_{X}\left(X\right),\rho ,r,N_{c}\right) & =-\frac{1}{N_{c}}\ln\int_{H}p_{H}\left(H\right)\left\{\int_{X^{\prime }}\int_{X}p_{X}\left(X\right)p_{X}\left(X^{\prime }\right)e^{r\left[\mathrm{tr}\left(XX^{†}\right)+\mathrm{tr}\left(X^{\prime }X^{\prime †}\right)-2N_{c}P\right]}\right.\\ \; & {\left.{\left[\int_{Y}\sqrt{p\left(Y|X,H\right)p\left(Y|X^{\prime },H\right)}dY\right]}^{1/\rho }dXdX^{\prime }\right\}}^{\rho }dH. \end{array}$$

The above Equation can be obtained as

(27) $$\begin{array}{cc} {\tilde{E}}_{x}\left(\rho ,\beta ,N_{c}\right) & =\underset{A^{\prime }\left(\rho ,\beta \right)}{\underset{︸}{2\rho \left(N_{t}-\beta \right)+2\rho N_{t}\ln\left(\frac{\beta }{N_{t}}\right)}}-\frac{1}{N_{c}}\ln\left(E\left\{\det{\left(I_{s}+\frac{\gamma }{2\rho \beta }\Theta \right)}^{-N_{c}\rho }\right\}\right) \end{array}$$

for the Gaussian input distribution and equal power allocation at the transmitter. Accordingly, the expurgated exponent in Equation (25) can be written as

(28) $$E_{ex}\left(p_{X}\left(X\right),R,N_{c}\right)=\max_{\rho \geq 1}\left(\max_{0\leq \beta \leq N_{t}}{\tilde{E}}_{x}\left(\rho ,\beta ,N_{c}\right)-\rho R\right).$$

## 3. Gallager’s Exponent for Gamma–Gamma Block Fading Channels

In this section, we present Gallager’s exponent’s upper bounds for coherent MIMO FSO systems over gamma–gamma fading channels. These results are established with the help of Hadamard inequality. Thus, it should be noted that the derived bounds are only mathematically meaningful. However, the analytical expressions of Gallager’s exponent are obtained for the MIMO FSO systems employing the STBC scheme, and the tightness of them is verified through the comparison with the exact results. The independent and identically distributed (i.i.d.) fading is considered here for convenience.

### 3.1. Random Coding Exponent Analysis

Using Hadamard inequality, we first investigate the random coding exponent and give the upper bound as follows.

**Proposition** **2.** *The random coding exponent for coherent MIMO FSO systems over gamma–gamma fading channels can be upper bounded by*

(29) $$\begin{array}{cc} E_{r}\left(R,N_{c}\right) & \leq {\bar{E}}_{r}\left(R,N_{c}\right)\\ \; & \leq \max_{0\leq \rho \leq 1}\left(\max_{0\leq \beta \leq N_{t}}A\left(\rho ,\beta \right)-\frac{s}{N_{c}}\ln\underset{g}{\underset{︸}{E\left\{{\left(1+\frac{\gamma \chi }{\beta (1+\rho )}\right)}^{-N_{c}\rho }\right\}}}-\rho R\right) \end{array}$$

*where χ denotes the sum of t statistically independent and identically distributed (i.i.d.) gamma–gamma variables. According to [22], it is known that a sum of t i.i.d. gamma–gamma variates with parameters (a,b,Ω) can be approximated efficiently by a single gamma–gamma distribution χ with parameters $\left(a_{t},b_{t},\Omega_{t}\right)$, where*

(30) $$\begin{array}{cc} a_{t} & =ta+\left(t-1\right)\frac{-0.127-0.95a-0.0058b}{1+0.00124a+0.98b}\\ b_{t} & =tb,\;\Omega_{t}=t\Omega \end{array}$$

*and*

(31) $$f\left(\chi \right)=\frac{2{\left(a_{t}b_{t}\right)}^{\frac{a_{t}+b_{t}}{2}}\chi^{\frac{a_{t}+b_{t}}{2}-1}}{\Gamma \left(a_{t}\right)\Gamma \left(b_{t}\right)\Omega_{t}^{\frac{a_{t}+b_{t}}{2}}}K_{a_{t}-b_{t}}\left[2\sqrt{\frac{a_{t}b_{t}}{\Omega_{t}}\chi }\right].$$

**Proof.**  A proof is given in Appendix B. □

The expectation expression in Equation (29) can be evaluated as

(32) $$\begin{array}{cc} g & =\int_{0}^{\infty }{\left(1+\frac{\gamma \chi }{\beta (1+\rho )}\right)}^{-N_{c}\rho }f\left(\chi \right)d\chi \\ \; & =\int_{0}^{\infty }\frac{2}{\Gamma \left(a_{t}\right)\Gamma \left(b_{t}\right)}{\left(1+\frac{\Omega_{t}\gamma y}{a_{t}b_{t}\beta \left(1+\rho \right)}\right)}^{-N_{c}\rho }y^{\frac{a_{t}+b_{t}}{2}-1}K_{a_{t}-b_{t}}\left[2\sqrt{y}\right]dy\\ \; & =\frac{2}{\Gamma \left(a_{t}\right)\Gamma \left(b_{t}\right)\Gamma \left(N_{c}\rho \right)}\int_{0}^{\infty }G_{1,1}^{1,1}\left({\left.\frac{\Omega_{t}\gamma y}{a_{t}b_{t}\beta \left(1+\rho \right)}\right|}_{0}^{1-N_{c}\rho }\right)y^{\frac{a_{t}+b_{t}}{2}-1}K_{a_{t}-b_{t}}\left[2\sqrt{y}\right]dy\\ \; & =\frac{1}{\Gamma \left(a_{t}\right)\Gamma \left(b_{t}\right)\Gamma \left(N_{c}\rho \right)}G_{3,1}^{1,3}\left({\left.\frac{\Omega_{t}\gamma }{a_{t}b_{t}\beta \left(1+\rho \right)}\right|}_{0}^{1-a_{t},1-b_{t},1-N_{c}\rho }\right) \end{array}$$

where in Equation (32), we have expressed ${\left(1+ax\right)}^{b}$ in terms of MeijerG function ([26], [(8.4.2.5)]) and used integration formula ([27], [(7.821.3)])

(33) $$\begin{array}{cc} \; & \int_{0}^{\infty }x^{-\rho }K_{v}\left(2\sqrt{x}\right)G_{p,q}^{m,n}\left({\left.\alpha x\right|}_{b_{1},\cdots ,b_{q}}^{a_{1},\cdots ,a_{p}}\right)dx=\frac{1}{2}G_{p+2,q}^{m,n+2}\left({\left.\alpha \right|}_{b_{1},\cdots ,b_{q}}^{\rho -\frac{1}{2}v,\rho +\frac{1}{2}v,a_{1},\cdots ,a_{p}}\right) \end{array}$$

if p+q<2(m+n), $\left|∠\alpha |<\right|\left(m+n-p/2-q/2\right)\pi |$, Re(ρ)<1−1/2|Re(v)|+min1≤j≤mRe(bj).

The derived upper bound involves the MeijerG function, which does not enable us to do further analysis. In the following, we derive a simpler expression for ${\bar{E}}_{r}\left(R,N_{c}\right)$ in the high SNR regime to gain more sight.

**Corollary** **1.** *For MIMO gamma–gamma fading channels using coherent detection, in the high SNR regime, the random coding exponent can be approximated as*

(34) $$\begin{array}{cc} {\bar{E}}_{r}\left(R,N_{c}\right) & \approx {\bar{E}}_{\mathrm{hsnr}}\left(R,N_{c}\right)\\ & \approx \max_{0\leq \rho \leq 1}\left(\max_{0\leq \beta \leq N_{t}}A\left(\rho ,\beta \right)-\frac{s}{N_{c}}\ln\left({\left(\frac{\beta \left(1+\rho \right)a_{t}b_{t}}{\gamma \Omega_{t}}\right)}^{N_{c}\rho }\frac{\Gamma \left(a_{t}-N_{c}\rho \right)\Gamma \left(b_{t}-N_{c}\rho \right)}{\Gamma \left(a_{t}\right)\Gamma \left(b_{t}\right)}\right)-\rho R\right). \end{array}$$

**Proof.**  At high SNRs, ${\left(1+\frac{\gamma \chi }{\beta (1+\rho )}\right)}^{-N_{c}\rho }$ reduces to ${\left(\frac{\gamma \chi }{\beta (1+\rho )}\right)}^{-N_{c}\rho }$. Then, we have

(35) $$\begin{array}{cc} g & ={\left(\frac{\beta (1+\rho )}{\gamma }\right)}^{N_{c}\rho }\int_{0}^{\infty }\chi^{-N_{c}\rho }f\left(\chi \right)d\chi \\ \; & ={\left(\frac{\beta \left(1+\rho \right)a_{t}b_{t}}{\gamma \Omega_{t}}\right)}^{N_{c}\rho }\frac{1}{\Gamma \left(a_{t}\right)\Gamma \left(b_{t}\right)4^{\frac{a_{t}+b_{t}}{2}-N_{c}\rho -1}}\int_{0}^{\infty }t^{a_{t}+b_{t}-2N_{c}\rho -1}K_{a_{t}-b_{t}}\left(t\right)dt\\ \; & ={\left(\frac{\beta \left(1+\rho \right)a_{t}b_{t}}{\gamma \Omega_{t}}\right)}^{N_{c}\rho }\frac{\Gamma \left(a_{t}-N_{c}\rho \right)\Gamma \left(b_{t}-N_{c}\rho \right)}{\Gamma \left(a_{t}\right)\Gamma \left(b_{t}\right)} \end{array}$$

where $\min\left(a_{t},b_{t}\right)>N_{c}\rho$ and the last equation holds in Equation (35) due to ([27], [(6.561.16)])

(36) $$\begin{array}{cc} \; & \int_{0}^{\infty }x^{\mu }K_{v}\left(ax\right)dx=2^{\mu -1}a^{-\mu -1}\Gamma \left(\frac{1+\mu +\upsilon }{2}\right)\Gamma \left(\frac{1+\mu -\upsilon }{2}\right). \end{array}$$

 □

**Corollary** **2.** *The upper bound of the random coding exponent in the high SNR regime, ${\bar{E}}_{hsnr}$, is a monotonic decreasing function of the channel coherence parameter $N_{c}$.*

**Proof.**  We prove the corollary by showing the first derivative of ${\bar{E}}_{hsnr}$ with respect to $N_{c}$ is strictly less than zero, which is given by

(37) $$\begin{array}{cc} \frac{d{\bar{E}}_{hsnr}}{dN_{c}} & =\frac{1}{N_{c}^{2}}\ln\frac{\Gamma \left(a_{t}-N_{c}\rho \right)\Gamma \left(b_{t}-N_{c}\rho \right)}{\Gamma \left(a_{t}\right)\Gamma \left(b_{t}\right)}+\frac{\rho }{N_{c}}\left(\psi \left(a_{t}-N_{c}\rho \right)+\psi \left(b_{t}-N_{c}\rho \right)\right)<0 \end{array}$$

where ψ(·) denotes the digamma function and is equivalent to

(38) $$\psi \left(x\right)=\left(-1\right)\sum_{k=0}^{\infty }\frac{1}{x+k}<0\;\mathrm{for}\;\mathrm{all}\;x>0$$

 □

**Figure 2:**
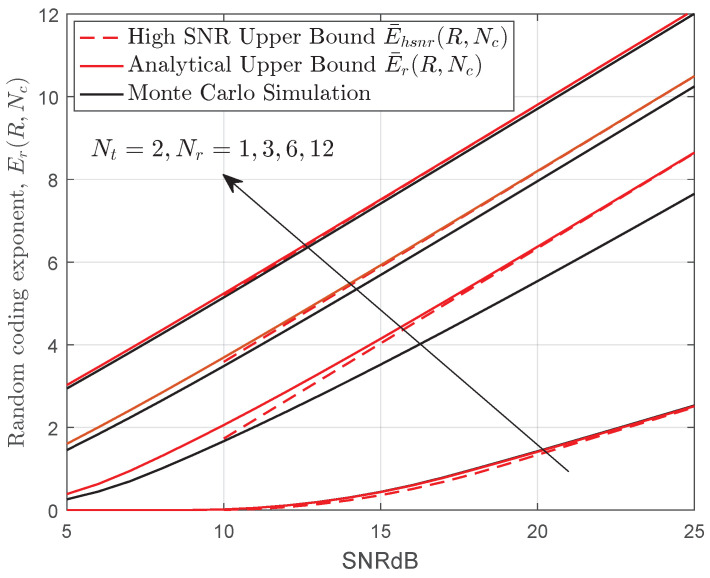
Monte Carlo simulation and upper bound on the random coding exponent for various MIMO systems when $a=2.94,b=2.59\;\left(C_{n}^{2}=2.638\times 10^{-14}\right)\;,\Omega =1,\;N_{c}=2$ and R=2 nats/symbol.

In Figure 2, we have plotted the random coding exponent for various MIMO systems. It can be seen that the upper bound becomes tighter with the increasing number of apertures and almost overlaps for t≫s, and this is due to

(39) $$\begin{array}{cc} \ln E\left\{\det{\left(I_{s}+a\Theta \right)}^{-N_{c}\rho }\right\} & \approx \ln E\left\{\prod_{l=1}^{s}{\left(1+a\Theta_{ll}\right)}^{-N_{c}\rho }\right\}\approx s\ln E\left\{{\left(1+a\Theta_{11}\right)}^{-N_{c}\rho }\right\}. \end{array}$$

for large *t*. Specifically, we found that the upper bound ${\bar{E}}_{r}\left(R,N_{c}\right)$ overlaps with the exact random coding exponent for the single-input multiple-output (SIMO) or multiple-input single-output (MISO) channel, namely, when s=1. Additionally, the upper bound ${\bar{E}}_{hsnr}\left(R,N_{c}\right)$ is also included in Figure 2 and gives a reasonable reference for ${\bar{E}}_{r}\left(R,N_{c}\right)$ in the high SNR regime.

**Figure 3:**
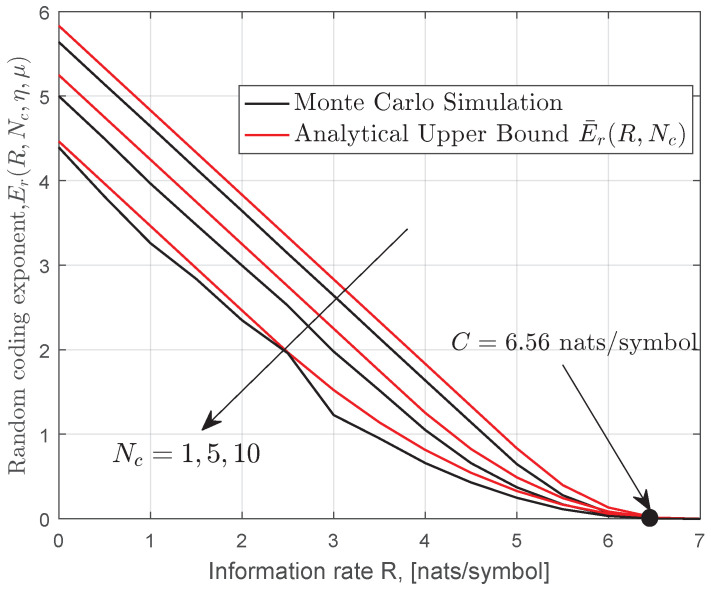
Monte Carlo simulation and upper bound on the random coding exponent for MIMO gamma–gamma fading channels when $N_{t}=2,N_{r}=6,a=2.94,b=2.59,\;\left(C_{n}^{2}=2.638\times 10^{-14}\right)\;,\Omega =1$ and γ = 10 dB.

In Figure 3, we investigate the effect of channel coherent time $N_{c}$ on the random coding exponent. It can be observed that the channel coherence time $N_{c}$ plays an important role in the error exponent. Note that the ergodic capacity with perfect CSI at the receiver is independent of $N_{c}$, which is consistent with the results shown in the literature.

Table 1 shows the required codeword length *L* for MIMO gamma–gamma fading channels with $N_{t}=2,N_{r}=8,\Omega =1,N_{c}=3$ at $P_{e}\leq 10^{-6}$. It is clear that there is a considerable reduction in the required codeword length from strong turbulence to weak turbulence. As expected, a higher SNR results in a shorter required codeword length for achieving the prescribed error probability $P_{e}$.

### 3.2. Ergodic Capacity Analysis

In this subsection, our focus is on the derivation of ergodic capacity for coherent MIMO FSO systems over gamma–gamma turbulence channel bases on Hadamard inequality.

**Proposition** **3.** *The ergodic capacity of MIMO gamma–gamma is upper bounded by*

(40) $$\langle C\rangle \leq \bar{C}=\frac{s}{\Gamma \left(a_{t}\right)\Gamma \left(b_{t}\right)}G_{4,2}^{1,4}\left({\left.\frac{\gamma \Omega_{t}}{a_{t}b_{t}N_{t}}\right|}_{1,0}^{1-a_{t},1-b_{t},1,1}\right)$$

**Proof.**  Similarly, using the Hadamard’s inequality, Equation (24) can be upper bounded by

(41) $$\begin{array}{cc} \langle C\rangle & \leq \bar{C}=sE\left\{\ln\left(1+\frac{\gamma }{N_{t}}\chi \right)\right\}\\ \; & \leq \frac{2s{\left(a_{t}b_{t}\right)}^{\frac{a_{t}+b_{t}}{2}}}{\Gamma \left(a_{t}\right)\Gamma \left(b_{t}\right)\Omega_{t}^{\frac{a_{t}+b_{t}}{2}}}\int_{0}^{\infty }\chi^{\frac{a_{t}+b_{t}}{2}-1}G_{2,2}^{1,2}\left({\left.\frac{\chi \gamma }{N_{t}}\right|}_{1,0}^{1,1}\right)K_{a_{t}-b_{t}}\left(2\sqrt{\frac{a_{t}b_{t}}{\Omega_{t}}\chi }\right)d\chi \\ \; & \leq \frac{s}{\Gamma \left(a_{t}\right)\Gamma \left(b_{t}\right)}G_{4,2}^{1,4}\left({\left.\frac{\gamma \Omega_{t}}{a_{t}b_{t}N_{t}}\right|}_{1,0}^{1-a_{t},1-b_{t},1,1}\right) \end{array}$$

where in Equation (41), we have expressed ln(1+ax) in terms of MeijerG function ([26], [(8.4.6.5)]) and used the integration formula Equation (33). □

To obtain further insights, a more simplified formula of capacity upper bound in the high SNR regime is presented.

**Corollary** **3.** *In the high SNR regime, the ergodic capacity upper bound $\bar{C}$ can be approximated as*

(42) $$\bar{C}\approx {\bar{C}}_{hsnr}=s\left(\ln\left(\frac{\gamma \Omega_{t}}{a_{t}b_{t}N_{t}}\right)+\psi \left(a_{t}\right)+\psi \left(b_{t}\right)\right).$$

**Proof.**  At high SNRs, $\ln\left(1+\frac{\gamma }{N_{t}}\chi \right)$ can be approximated by $\ln\left(\frac{\gamma }{N_{t}}\chi \right)$ and we have

(43) $$\begin{array}{cc} \bar{C}\approx {\bar{C}}_{hsnr} & =\int_{0}^{\infty }\frac{s2{\left(a_{t}b_{t}\right)}^{\frac{a_{t}+b_{t}}{2}}}{\Gamma \left(a_{t}\right)\Gamma \left(b_{t}\right)\Omega_{t}^{\frac{a_{t}+b_{t}}{2}}}\ln\left(\frac{\gamma \chi }{N_{t}}\right)\chi^{\frac{a_{t}+b_{t}}{2}-1}K_{a_{t}-b_{t}}\left(2\sqrt{\frac{a_{t}b_{t}}{\Omega_{t}}\chi }\right)d\chi \\ \; & =\int_{0}^{\infty }\frac{s}{\Gamma \left(a_{t}\right)\Gamma \left(b_{t}\right)2^{a_{t}+b_{t}-2}}\left(\ln\left(\frac{\gamma \Omega_{t}}{4a_{t}b_{t}N_{t}}k^{2}\right)\right)k^{a_{t}+b_{t}-1}K_{a_{t}-b_{t}}\left(k\right)dk\\ \; & =s\left(\ln\left(\frac{\gamma \Omega_{t}}{a_{t}b_{t}N_{t}}\right)+\psi \left(a_{t}\right)+\psi \left(b_{t}\right)\right) \end{array}$$

(44) $$\begin{array}{cc} \int_{0}^{\infty }\ln\left(t\right)t^{s-1}K_{v}\left(t\right)dt & \overset{\left(a\right)}{=}\frac{\sqrt{\pi }}{2^{v}\Gamma \left(v+1/2\right)}\int_{0}^{1}x^{-2v-1}{\left(1-x^{2}\right)}^{v-1/2}\int_{0}^{\infty }t^{s+v-1}e^{-t/x}\ln\left(t\right)dtdx\\ \; & \overset{\left(b\right)}{=}\frac{\sqrt{\pi }}{2^{v}\Gamma \left(v+1/2\right)}\int_{0}^{1}x^{s-v-1}{\left(1-x^{2}\right)}^{v-1/2}\Gamma \left(v+s\right)\left[\psi \left(v+s\right)+\ln\left(x\right)\right]dx\\ \; & \overset{\left(c\right)}{=}2^{s-2}\Gamma \left(\frac{s+v}{2}\right)\Gamma \left(\frac{s-v}{2}\right)\left(2\psi \left(v+s\right)+\psi \left(\frac{s-v}{2}\right)-\psi \left(\frac{s+v+1}{2}\right)\right)\\ \; & \overset{\left(d\right)}{=}2^{s-3}\Gamma \left(\frac{s+v}{2}\right)\Gamma \left(\frac{s-v}{2}\right)\left(\psi \left(\frac{s-v}{2}\right)+\psi \left(\frac{s+v}{2}\right)+\ln4\right) \end{array}$$

In deriving the equation above, we have used the relations shown in Equation (44), where we have used ([28], (Example 3.11)) for (a), ([27], (4.352.1)) for (b), ([27], (4.253.1) and (3.197.4)), ([28], (5.7)) for (c) and ([28], (8.370) and (8.377)) for (d). □

The above corollary reveals that the effects of small and large-scale fading are decoupled in the high SNR regime, which is consistent with the results shown for the Nakagami channels ([29], (Corollary 5)).

**Corollary** **4.** *The ergodic capacity upper bound approximation, ${\bar{C}}_{hsnr}$, is a monotonic increasing function of the channel fading parameter a.*

**Proof.**  It is easy to show that the first derivative of ${\bar{C}}_{hsnr}$ with respect to *a* is greater than zero and this is done as follows:

(45) $$\begin{array}{cc} \frac{d{\bar{C}}_{hsnr}}{da} & =s\left[\psi^{\left(1\right)}\left(a_{t}\right)-\frac{1}{a_{t}}\right]\frac{da_{t}}{da}\\ \; & =s\frac{da_{t}}{da}\left[\sum_{k=0}^{\infty }\frac{1}{{\left(a_{t}+k\right)}^{2}}-\frac{1}{a_{t}}\right]\\ \; & >s\frac{da_{t}}{da}\left[\sum_{k=0}^{\infty }\frac{1}{\left(a_{t}+k\right)\left(a_{t}+k+1\right)}-\frac{1}{a_{t}}\right]\\ \; & >s\frac{da_{t}}{da}\left[\sum_{k=0}^{\infty }\left(\frac{1}{\left(a_{t}+k\right)}-\frac{1}{\left(a_{t}+k+1\right)}\right)-\frac{1}{a_{t}}\right]\\ \; & >0. \end{array}$$

 □

**Figure 4:**
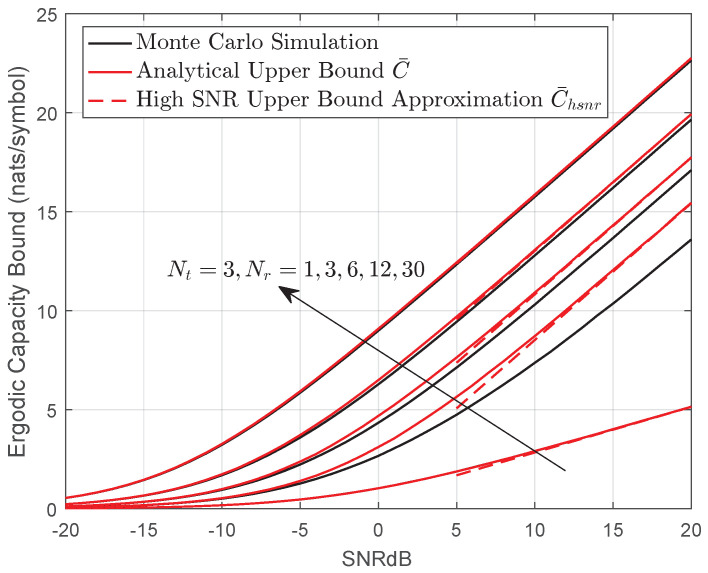
Analytical upper bound, simulated and high-SNR approximation ergodic capacity for MIMO systems over gamma–gamma fading channel when $a=2.7,b=2.306\left(C_{n}^{2}=3.1\times 10^{-14}\right),\Omega =2$.

**Figure 5:**
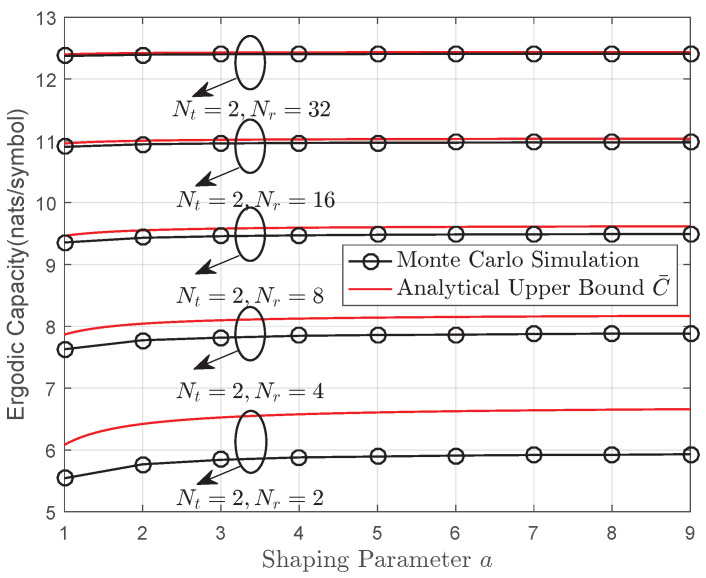
Ergodic capacity of the MIMO gamma–gamma fading channel: analytical upper versus Monte Carlo simulation results with shaping parameters *a* and $N_{r}$, where b=2.8,Ω=1, γ=15 dB.

Figure 4 presents the monte carlo simulation, analytical expression Equation (40) and high-SNR approximation Equation (42) ergodic capacity results for various MIMO systems. It can be seen that the upper bound $\bar{C}$ provides reasonable reference to the actual performance for a large MIMO system. In addition, the derived bound $\bar{C}$ shows the exact capacity results for a SIMO or MISO channel when s=1. The same conclusion can be also drawn from Figure 5. Note that for a fixed transmit aperture $N_{t}$, increasing number of receiver apertures $N_{r}$ helps overcome the effect of fading. For instance, when $N_{r}=2$, the ergodic capacity increases considerably when *a* ranges from 1 to 9. However, the difference is almost inappreciable for $N_{r}=32$.

### 3.3. Expurgated Exponents

The expurgated exponent for MIMO FSO systems is considered in this subsection. Thus, we have

**Proposition** **4.** *The expurgated exponent for coherent MIMO FSO systems over gamma–gamma fading channels can be upper bounded by*

(46) $$\begin{array}{cc} E_{ex}\left(p_{X}\left(X\right),R,N_{c}\right) & \leq {\bar{E}}_{ex}\left(p_{X}\left(X\right),R,N_{c}\right)\\ \; & \leq \max_{\rho \geq 1}\left(\max_{0\leq \beta \leq N_{t}}A^{\prime }\left(\rho ,\beta \right)-\rho R-\frac{s}{N_{c}}\ln\frac{G_{3,1}^{1,3}\left.{\left(\frac{\Omega_{t}\gamma }{2a_{t}b_{t}\beta \rho }\right|}_{0}^{1-a_{t},1-b_{t},1-N_{c}\rho }\right)}{\Gamma \left(a_{t}\right)\Gamma \left(b_{t}\right)\Gamma \left(N_{c}\rho \right)}\right). \end{array}$$

**Proof.**  The proof follows a similar line of reasoning as in Proposition 2. □

**Corollary** **5.** *At high SNRs, the above bound ${\bar{E}}_{ex}\left(p_{X}\left(X\right),R,N_{c}\right)$ reduces to*

(47) $$\begin{array}{cc} & {\bar{E}}_{ex}\left(p_{X}\left(X\right),R,N_{c}\right)\approx \max_{\rho \geq 1}\left(\max_{0\leq \beta \leq N_{t}}A^{\prime }\left(\rho ,\beta \right)-\rho R-\frac{s}{N_{c}}\ln\left[{\left(\frac{2\beta \rho a_{t}b_{t}}{\gamma \Omega_{t}}\right)}^{N_{c}\rho }\frac{\Gamma \left(a_{t}-N_{c}\rho \right)\Gamma \left(b_{t}-N_{c}\rho \right)}{\Gamma \left(a_{t}\right)\Gamma \left(b_{t}\right)}\right]\right). \end{array}$$

**Proof.**  The proof follows a similar line of reasoning as in Corollary 3 and is omitted here. □

**Figure 6:**
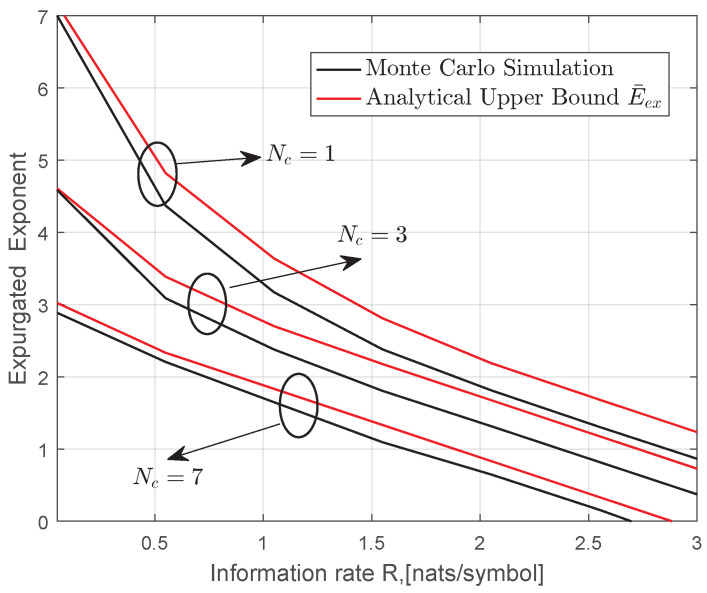
Monte Carlo simulation and upper bound of the expurgated exponent for MIMO gamma–gamma fading channels when $N_{t}=2,N_{r}=3$, γ=10dB, a=2.7,b=2.306$\left(C_{n}^{2}=3.1\times 10^{-14}\right)$, Ω=1.

In Figure 6, the expurgated exponent is plotted as a function of *R* for different coherence time over strong turbulence channel. As expected, system performance becomes worse with increasing coherence time $N_{c}$.

## 4. Error Exponent for MIMO-STBC Systems

It has been shown in Section 2 that the MIMO systems reduce to SISO systems when employing the STBC technique and let

(48) $$\Xi =\sum_{i=1}^{N_{r}N_{t}}|h_{i}{|}^{2}=\sum_{i=1}^{st}{\left|h_{i}\right|}^{2}.$$

Thus, the probability density function (pdf) of Ξ follows gamma–gamma distribution with parameters $\left(a_{st},b_{st},\Omega_{st}\right)$, which is given by

(49) $$p_{\Xi }\left(z\right)=\frac{2{\left(a_{st}b_{st}\right)}^{\frac{a_{st}+b_{st}}{2}}z^{\frac{a_{st}+b_{st}}{2}-1}}{\Gamma \left(a_{st}\right)\Gamma \left(b_{st}\right)\Omega_{t}^{\frac{a_{st}+b_{st}}{2}}}K_{a_{st}-b_{st}}\left[2\sqrt{\frac{a_{st}b_{st}}{\Omega_{st}}z}\right].$$

Accordingly,

(50) $$\begin{array}{cc} a_{st} & =sta+\left(st-1\right)\frac{-0.127-0.95a-0.0058b}{1+0.00124a+0.98b}\\ b_{st} & =stb,\;\Omega_{t}=st\Omega \end{array}$$

(1) *STBC random coding exponent*: Note that Equation (20) can be simplified into

(51) $${\tilde{E}}_{0,STBC}=A\left(\rho ,\beta \right)-\frac{1}{N_{c}}\ln\left(E\left\{{\left(1+\frac{\gamma \Xi }{\beta (1+\rho )}\right)}^{-N_{c}\rho }\right\}\right).$$

**Proposition** **5.** *The random coding exponent $E_{r,STBC}$ for MIMO STBC systems can be derived as*

(52) $$\begin{array}{cc} E_{r,STBC}\left(R,N_{c}\right) & =\max_{0\leq \rho \leq 1}\left(\max_{0\leq \beta \leq N_{t}}A\left(\rho ,\beta \right)-\rho R-\frac{1}{N_{c}}\ln\frac{G_{3,1}^{1,3}\left({\left.\frac{\Omega_{st}\gamma }{a_{st}b_{st}\beta \left(1+\rho \right)}\right|}_{0}^{1-a_{st},1-b_{st},1-N_{c}\rho }\right)}{\Gamma \left(a_{st}\right)\Gamma \left(b_{st}\right)\Gamma \left(N_{c}\rho \right)}\right). \end{array}$$

According to the ([29], (Example 2)), Equation (52) can be regarded as a lower bound of the $E_{r}\left(R,N_{c}\right)$, namely, $E_{r,STBC}\left(R,N_{c}\right)\leq E_{r}\left(R,N_{c}\right)$.

**Proof.**  The proof follows a similar line of reasoning as in Proposition 2 and is omitted here. □

(2) *STBC Ergodic Capacity*:

**Corollary** **6.** *The ergodic capacity of STBC over MIMO gamma–gamma fading channels can be expressed as*

(53) $$C_{STBC}=\frac{1}{\Gamma \left(a_{st}\right)\Gamma \left(b_{st}\right)}G_{4,2}^{1,4}\left({\left.\frac{\gamma \Omega_{st}}{a_{st}b_{st}N_{t}}\right|}_{1,0}^{1-a_{st},1-b_{st},1,1}\right).$$

**Proof.**  The proof follows a similar line of reasoning as in Proposition 3. □

(3) *STBC expurgated exponent*:

**Corollary** **7.** *The expurgated exponent of STBC over gamma–gamma MIMO fading channels can be obtained as*

(54) $$\begin{array}{cc} E_{ex,STBC} & =\max_{\rho \geq 1}\left(\max_{0\leq \beta \leq N_{t}}A^{\prime }\left(\rho ,\beta \right)-\rho R-\frac{1}{N_{c}}\ln\frac{G_{3,1}^{1,3}\left({\left.\frac{\Omega_{st}\gamma }{2a_{st}b_{st}\beta \rho }\right|}_{0}^{1-a_{st},1-b_{st},1-N_{c}\rho }\right)}{\Gamma \left(a_{st}\right)\Gamma \left(b_{st}\right)\Gamma \left(N_{c}\rho \right)}\right). \end{array}$$

Then, in order to obtain additional insights for $E_{r,STBC}$, $C_{STBC}$ and $E_{ex,STBC}$, we now elaborate on the high-SNR regime and have

(55) $$\begin{array}{cc} & E_{r,STBC,hsnr}=\max_{0\leq \rho \leq 1}\left(\max_{0\leq \beta \leq N_{t}}A\left(\rho ,\beta \right)-\rho R-\frac{1}{N_{c}}\ln\left({\left(\frac{\beta \left(1+\rho \right)a_{st}b_{st}}{\gamma \Omega_{st}}\right)}^{N_{c}\rho }\frac{\Gamma \left(a_{st}-N_{c}\rho \right)\Gamma \left(b_{st}-N_{c}\rho \right)}{\Gamma \left(a_{st}\right)\Gamma \left(b_{st}\right)}\right)\right)\\ & {\langle C\rangle }_{STBC,hsnr}=\left(\ln\left(\frac{\gamma \Omega_{st}}{a_{st}b_{st}N_{t}}\right)+\psi \left(a_{st}\right)+\psi \left(b_{st}\right)\right)\\ & E_{ex,STBC,hsnr}=\max_{\rho \geq 1}\left(\max_{0\leq \beta \leq N_{t}}A^{\prime }\left(\rho ,\beta \right)-\rho R-\frac{1}{N_{c}}\ln\left[{\left(\frac{2\beta \rho a_{st}b_{st}}{\gamma \Omega_{t}}\right)}^{N_{c}\rho }\frac{\Gamma \left(a_{st}-N_{c}\rho \right)\Gamma \left(b_{st}-N_{c}\rho \right)}{\Gamma \left(a_{st}\right)\Gamma \left(b_{st}\right)}\right]\right). \end{array}$$

**Figure 7:**
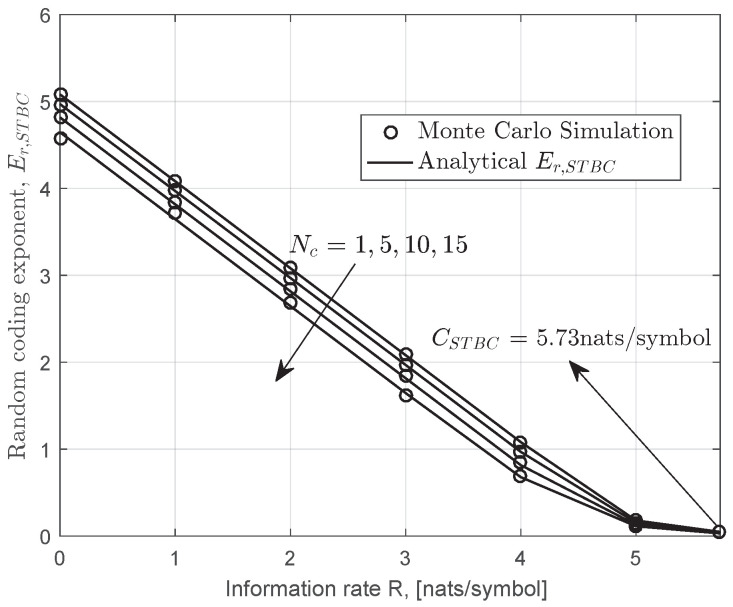
Monte Carlo simulation and analytical random coding exponent for space-time block code (STBC) systems over gamma–gamma fading channels when $N_{t}\;=\;N_{r}\;=\;4,a\;=\;2.94,b\;=\;2.59\;\left(C_{n}^{2}\;=\;2.64\times 10^{-14}\right),\;\Omega =2.5$.

In Figure 7, we present the results of the random coding exponent of STBC over the strong turbulence channel; the analytical results were derived according to Equation (52). It can be seen that the random coding exponent decreases monotonically with the parameter $N_{c}$. In other words, it is impossible to transmit information at a positive rate with arbitrarily small error probability when $N_{c}\to \infty$. As expected, the ergodic capacity is independent of coherence time $N_{c}$.

**Figure 8:**
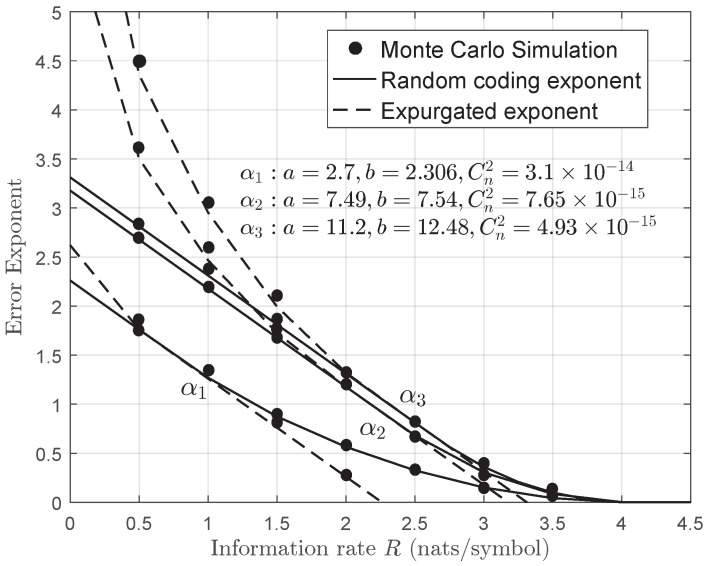
Monte Carlo simulation and analytical random coding exponent for STBC systems over gamma–gamma fading channels when $N_{t}=N_{r}=4,a=2.94,b=2.59\;\left(C_{n}^{2}=2.64\times 10^{-14}\right),\;\Omega =2.5$.

In Figure 8, we have plotted the random coding exponent and expurgated exponent under turbulence strengths. It can be observed that there is a performance improvement as both shaping parameters a,b increase, i.e., from strong turbulence to weak turbulence channels, which indicates a shorter code is required to achieve the same level of reliable communications. The same conclusion can be also drawn in Table 2.

## 5. Conclusions

In this paper, a detailed Gallager’s exponent analysis for the coherent MIMO FSO systems was presented in order to investigate the fundamental tradeoff between communication reliability and information rate. In particular, we considered gamma–gamma fading channels, which have been exhaustively used in the performance analysis of FSO communication systems. For the considered models, the upper bounds of the random coding exponent, ergodic capacity and expurgated exponent were derived by virtue of Hadamard inequality, which allows us to avoid calculating the eigenvalue distribution of the channel matrix. Moreover, in the high SNR regime, we have derived simple closed-form expressions of upper bounds to gain further insights into the effects of the system parameters, including shaping parameter *a* and the number of apertures $N_{t},N_{r}$. Note that the effects of small- and large-scale were found to be decoupled for the ergodic capacity upper bound at high SNRs. The performance metrics for MIMO FSO systems employing the STBC scheme were also investigated. We noticed that larger values of a,b tend to increase Gallager’s exponent or communication reliability.

## Figures and Tables

**Table 1:** Required codeword lengths *L* over gamma–gamma fading channels at rate R=9 nats/symbol with $P_{e}\leq 10^{-6},N_{t}=2,N_{r}=8,\Omega =1$ and $N_{c}=3$.

**SNRdB** γ **= 15 dB**	**Weak Turbulence** $C_{n}^{2}=4.93\times 10^{-15}$	**Moderate Turbulence** $C_{n}^{2}=7.65\times 10^{-15}$	**Strong Turbulence** $C_{n}^{2}=3.1\times 10^{-14}$
Description	a=2.7 b=2.306	a=7.49 b=7.54	a=11.2 b=12.48
Exact	435	270	240
Lower Bound	342	207	192
**SNRdB** γ **= 16 dB**	**Weak Turbulence** $C_{n}^{2}=4.93\times 10^{-15}$	**Moderate Turbulence** $C_{n}^{2}=7.65\times 10^{-15}$	**Strong Turbulence** $C_{n}^{2}=3.1\times 10^{-14}$
Description	a=2.7 b=2.306	a=7.49 b=7.54	a=11.2 b=12.48
Exact	120	78	75
Lower Bound	105	69	63
**SNRdB** γ **= 17 dB**	**Weak Turbulence** $C_{n}^{2}=4.93\times 10^{-15}$	**Moderate Turbulence** $C_{n}^{2}=7.65\times 10^{-15}$	**Strong Turbulence** $C_{n}^{2}=3.1\times 10^{-14}$
Description	a=2.7 b=2.306	a=7.49 b=7.54	a=11.2 b=12.48
Exact	54	36	36
Lower Bound	48	33	30

**Table 2:** Required codeword lengths *L* over gamma–gamma fading channels at rate R=4.5 nats/symbol with $P_{e}\leq 10^{-6},N_{t}=2,N_{r}=2,\Omega =2.5$ and $N_{c}=5$.

	Strong Turbulence $C_{n}^{2}=3.1\times 10^{-14}$	Moderate Turbulence $C_{n}^{2}=7.65\times 10^{-15}$	Weak Turbulence $C_{n}^{2}=4.93\times 10^{-15}$
SNRdB γ	a=2.7 b=2.306	a=7.49 b=7.54	a=11.2 b=12.48
14	2065	800	670
15	530	250	215
17.5	100	55	50
20	45	25	20

## Appendix A

According to Equation (19), a sufficient equal power allocation can maximize both

(A1) $$\ln\det\left(I_{n_{T}}-rQ\right)$$

and

(A2) $$-\frac{1}{N_{c}}\ln E\left\{\det{\left(I_{n_{R}}+\frac{H{\left(Q^{-1}-rI_{n_{T}}\right)}^{-1}H^{†}}{N_{0}\left(1+\rho \right)}\right)}^{-N_{c}\rho }\right\}$$

expressions. For Equation (A1), we find $I_{N_{t}}-rQ$ should be positive definite for a meaningful result in Equation (A1) and can then be diagonalized by unitary U, i.e., $I_{N_{t}}-rQ=U\Lambda U^{†}$, where $\Lambda =\mathrm{diag}\left(1-r\lambda_{1},\cdots ,1-r\lambda_{N_{t}}\right)$ and $1-\lambda_{1},\cdots ,1-\lambda_{N_{t}}\geq 0$. Thus

(A3) $$\det\left(I_{N_{t}}-rQ\right)=\prod_{l=1}^{N_{t}}\left(1-r\lambda_{l}\right).$$

Then we formulate an optimization problem under a power constraint

(A4) $$\begin{array}{cc} \; & \max_{\lambda_{1},\cdots ,\lambda_{N_{t}}}\prod_{l=1}^{N_{t}}\left(1-r\lambda_{l}\right)\\ \; & \;s.t.\;\sum_{l=1}^{N_{t}}\lambda_{l}\leq P. \end{array}$$

A Lagrange multiplier method can be employed to solve Equation (A4), which is expressed as

(A5) $$L\left(\lambda_{1},\cdots ,\lambda_{N_{t}};\alpha \right)=\prod_{l=1}^{N_{t}}\left(1-r\lambda_{l}\right)+\alpha \left(\sum_{l=1}^{N_{t}}\lambda_{l}-P\right).$$

Then, we have

(A6) $$\lambda_{1}=\lambda_{2}=\cdots =\lambda_{N_{t}}=\frac{P}{N_{t}}.$$

It can be observed that maximization of Equation (A2) can be equivalent to optimizing

(A7) $$\max_{Q}\det\left(I_{N_{r}}+\frac{H{\left(Q^{-1}-rI_{N_{t}}\right)}^{-1}H^{†}}{N_{0}\left(1+\rho \right)}\right).$$

For convenience, we define $M={\left(Q^{-1}-rI_{N_{t}}\right)}^{-1}$. Note that the matrix M is also symmetric, of which the eigenvalues are $\left[\frac{\lambda_{1}}{1-\lambda_{1}r},\cdots ,\frac{\lambda_{N_{t}}}{1-\lambda_{N_{t}}r}\right]\geq 0$. Using the relation $\det\left(I+AB\right)=\det\left(I+BA\right)$, the matrix $HMH^{†}$ must be positive definite according to

(A8) $$\det\left(HMH^{†}\right)=\det\left(MH^{†}H\right)=\det\left(M\right)\det\left(H^{†}H\right).$$

Thus, by the singular value decomposition theorem for $H=U\Sigma V^{†}$, we have

(A9) $$\begin{array}{cc} \det\left(I_{N_{t}}+\frac{H^{†}MH}{N_{0}\left(1+\rho \right)}\right) & =\det\left(I_{N_{r}}+\frac{DUMU^{†}D}{N_{0}\left(1+\rho \right)}\right)\leq \prod_{l=1}^{N_{t}}\left(1+\frac{\sigma_{l}^{2}{\left(UMU^{†}\right)}_{ll}}{N_{0}\left(1+\rho \right)}\right) \end{array}$$

where $\Sigma =\mathrm{diag}\left[\sigma_{1},\cdots ,\sigma_{N_{t}}\right]$. Specifically, equality holds for Equation (A9) when $UMU^{†}$ is diagonal, which indicates Q should be diagonal. Therefore, based on the above argument, Equation (19) can be maximized with $Q=\frac{P}{N_{t}}I_{N_{t}}$.

## Appendix B

Note that for any non-negative definite matrix $A\in C^{n\times n}$, the following inequality holds:

(A10) $$\det\left(A\right)\leq \prod_{i=1}^{n}A_{ii}$$

which is also known as Hadamard’s determinantal inequality [30]. Thus, we have

(A11) $$\ln E\left\{\det{\left(I_{s}+\frac{\gamma \Theta }{\beta (1+\rho )}\right)}^{-N_{c}\rho }\right\}\geq \frac{s}{N_{c}}\ln E\left\{{\left(1+\frac{\gamma \chi }{\beta (1+\rho )}\right)}^{-N_{c}\rho }\right\}$$

where χ denotes the sum of *t* i.i.d. gamma–gamma random variates. By substituting Equation (A11) into Equation (21), Equation (29) is then obtained. This completes the proof.
